# Isolated Splenic Vein Thrombosis: 8-Year-Old Boy with Massive Upper Gastrointestinal Bleeding and Hypersplenism

**DOI:** 10.1155/2015/480507

**Published:** 2015-08-05

**Authors:** Mohammad Ali Kiani, Arash Forouzan, Kambiz Masoumi, Behnaz Mazdaee, Mohammad Bahadoram, Hamid Reza Kianifar, Hassan Ravari

**Affiliations:** ^1^Pediatric Gastroenterology Ward, Department of Pediatrics, Mashhad University of Medical Sciences, Mashhad 9177948564, Iran; ^2^Department of Emergency Medicine, Imam Khomeini General Hospital, Ahvaz Jundishapur University of Medical Sciences, Ahvaz 6193673166, Iran; ^3^Medical Student Research Committee and Social Determinant of Health Research Center, Ahvaz Jundishapur University of Medical Sciences, Ahvaz 6193673166, Iran; ^4^Vascular and Endovascular Surgery Research Center, Imam Reza Hospital, Faculty of Medicine, Mashhad University of Medical Sciences, Mashhad 9177948564, Iran

## Abstract

We present an 8-year-old boy who was referred to our center with the complaint of upper gastrointestinal bleeding and was diagnosed with hypersplenism and progressive esophageal varices. Performing a computerized tomography (CT) scan, we discovered a suspicious finding in the venography phase in favor of thrombosis in the splenic vein. Once complementary examinations were done and due to recurrent bleeding and band ligation failure, the patient underwent splenectomy. And during the one-year follow-up obvious improvement of the esophageal varices was observed in endoscopy.

## 1. Introduction

**Figure 1:**
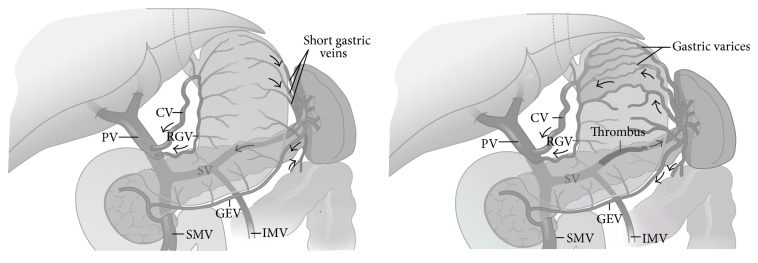
The effects of splenic vein thrombosis on normal venous anatomy. Note the gastric varices, dilatation of short gastric, and gastroepiploic (GEV) and coronary (CV) veins. The portal vein (PV), superior mesenteric vein (SMV), and inferior mesenteric vein (IMV) are patent. RGV: right gastric vein; SV: splenic vein [6].

Splenic vein thrombosis (SVT) is known as one of the rare causes of upper gastrointestinal bleeding and is mostly seen in the fifth decade of life and male sex [1, 2]. So far, 37 different etiologies have been reported for SVT [3]. The typical manifestations are bleeding from gastric varices characterized by anemia, hematemesis, melena, or hematochezia seen in 15%–50% of patients. Splenomegaly is observed in all patients with SVT whether it be in physical examination or imaging evaluation or during surgery [3]. Sometimes other symptoms of splenomegaly such as thrombocytopenia or pancytopenia and abdominal pain are also manifestations of the disease [4]. It must be noted that since liver cirrhosis is absent in SVT, these patients do not have chronic liver disease [4]. Being asymptomatic, in most cases, makes the diagnosis of SVT difficult. However reports of SVT have increased in recent years which could be due to advancement in imaging techniques [5]. Previously, though, most cases of SVT were distinguished in postmortem autopsy but now with the developments such as celiac angiography and splenoportography most cases are readily detectable. Since abdominal CT is used for patients with acute pancreatitis as well as preoperative patients of chronic pancreatitis, SVT is often an incidental finding in the computerized tomography (CT) scan [5]. The diagnosis test of choice for the assessment of SVT is venous-phase celiac angiography [5]. This condition results in increased localized sinistral portal pressure, which is also known as sinistral portal hypertension. The majority of patients with SVT and the resulting sinistral portal hypertension, in contrast to patients with generalized portal hypertension, are asymptomatic and have normal hepatic function. Gastrointestinal bleeding secondary to esophageal or gastric varices may occur in these patients (Figure 1). Most patients with SVT have peripheral arteries other than gastric varices which never go bleeding. As a result asymptomatic patients without gastric varices should be monitored and do not need treatment for SVT [4]. In other words SVT alone does not need treatment. In symptomatic patients splenectomy is the treatment of choice done by shunting collateral flow [4].

## 2. Case Report

**Figure 2:**
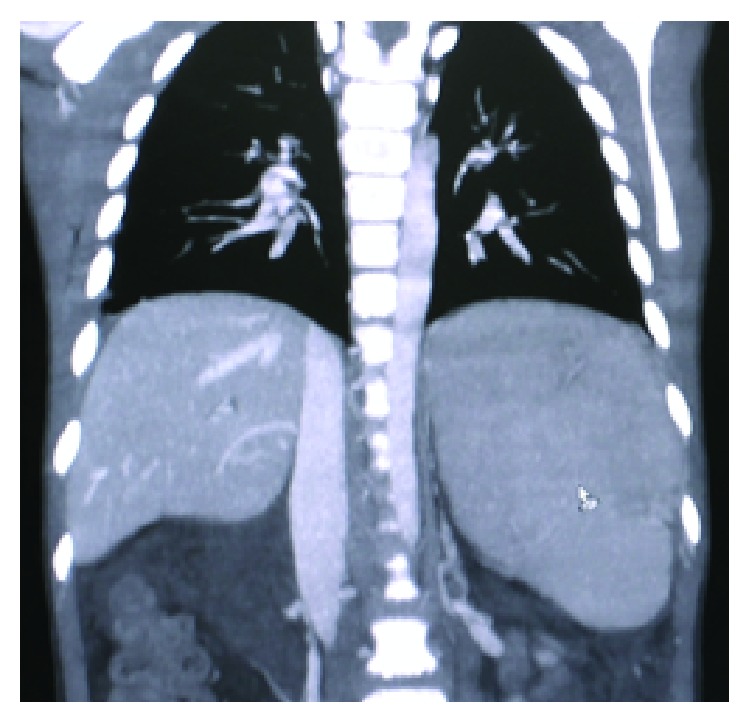
Patient's CT angiography of the abdomen revealed splenomegaly.

**Figure 3:**
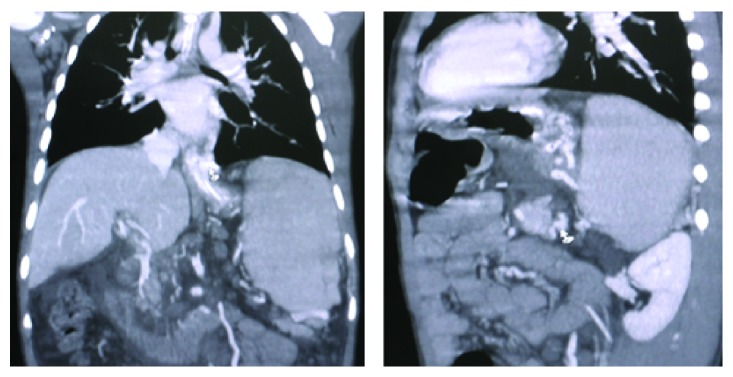
Dilation and tortuosity of spleen hilum veins and veins lining the esophagus and stomach.

**Figure 4:**
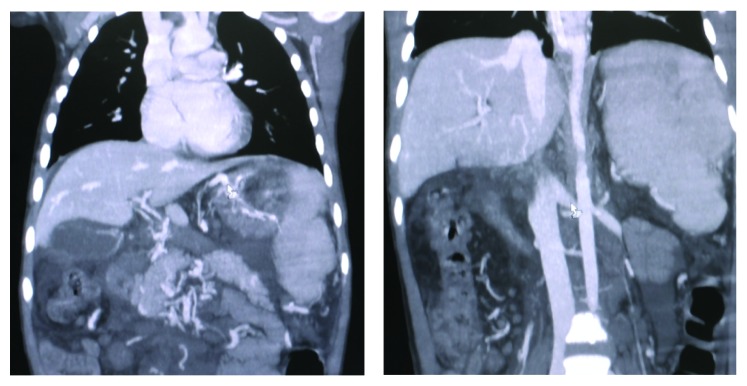
Dilation of coronary and left renal veins.

Patient was an 8-year-old boy referred to our center with massive upper gastrointestinal bleeding. The patient was found to have splenomegaly. He went through multiple evaluations including endoscopy and underwent band ligation for progressive esophageal varices. The patient had no history of neonatal blood exchange and umbilical venous catheters during infancy. No finding suggestive of portal venous thrombosis was observed in Doppler sonography. All the experiments indicated normal hepatic function. Due to recurrent bleeding and signs of hypersplenism and bicytopenia (thrombocytopenia and anemia), CT scan in venography phase was performed, which presented suspicious findings in splenic vein. Patient's CT angiography of the abdomen revealed splenomegaly (Figure 2), dilation and tortuosity of spleen hilum veins and veins lining the esophagus and stomach (Figure 3), and dilation of coronary and left renal veins (Figure 4). Haziness was observed throughout the mesenteric fat of the spleen hilum and splenic vein pathway. However, the size and density of the pancreas were normal. Additionally, lipase and amylase levels were also normal both in initial evaluations and in subsequent follow-ups. Given the portal hypertension symptoms and the history of cytopenia and splenic vein thrombosis, complementary evaluations were made considering PNH. Flow cytometry of peripheral blood was carried out on white blood cells in which CD55 and CD59 were reported as 90% positive. Gallbladder as well as intra- and extrahepatic biliary ducts were seen as normal. Splenorenal shunt was observable through left renal vein and splenogastric shunt was visible because of the tortuosity and dilation of gastric veins and enlargement of coronary vein. Regarding the clinical status of the patient, such as recurrent bleeding and failure to respond to band ligation in the specified time, the patient was scheduled for surgery. He underwent laparotomy under general anesthesia and in sterile conditions. The laparotomy findings were as follows: normal liver in inspection and palpation. There were plenty of omental adhesion bands surrounding the spleen. The spleen was larger than usual. Dilated veins surrounding the spleen and stomach and esophagus were observed. To release adhesions surrounding the spleen, the splenic vessels ligations were cut. Splenectomy was completed. It is worth noting that, due to signs of hypersplenism and the presence of splenic thrombosis, diagnostic assessments were done prior to surgery on MPS including ET and PV. JAK2 mutation was checked and reported as negative. Aspiration and bone marrow biopsy were also performed which reported bone marrow as normocellular and reactive. During the one-year follow-up the patient did not go into relapse and control endoscopies showed improvement of esophageal varices. In the assessments that followed hereditary deficits of pro S, pro C and Antithrombin III were checked for and reported as negative. The normal values are added to Table 1.

## 3. Discussion

Portal hypertension resulting from SVT can lead to massive gastrointestinal bleeding from esophageal or gastric varices or cause hypertensive gastropathy [1]. In 7%–20% of patients SVT is accompanied by acute and chronic pancreatitis, pancreatic pseudocysts, and pancreatic adenocarcinoma [1, 2]. Even after recovery, symptoms of pancreatitis have been reported [1, 4]. The most common cause of SVT is chronic pancreatitis and perivenous inflammation [4]. Although SVT has been reported in more than 45% of patients with chronic pancreatitis, most of SVT patients are asymptomatic [3]. Pancreatitis leading to SVT may be mild and the patients show no clinical sign indicating chronic pancreatitis [3].

Unlike patients with portal vein hypertension, most patients with SVT are asymptomatic and have normal hepatic function [4]. SVT can cause localized hypertension of splenic veins and create collaterals from spleen to the fundus. From that point, blood returns to the main portal system via coronary vein. In such a case, gastric varices are not often associated with esophageal varices except for collaterals at the site of gastroesophageal junction, where bleeding is common. In other cases, despite the formation of large and multiple collaterals, spontaneous bleeding seldom occurs.

Patients with the following characteristics are suspected of having SVT [4]: patients with history of pancreatitis or GI bleeding, patients with splenomegaly without portal vein hypertension, cirrhosis, or hematologic disease, and finally patients with gastric varices alone. There are other reasons for gastric bleeding in patients with chronic pancreatitis which include the following: arterial pseudoaneurysm, pancreatic pseudocysts, hemosuccus pancreaticus, peptic ulcer disease, gastritis, and Mallory-Weiss tears [7].

Therefore, an overall assessment in patients with SVT and gastrointestinal bleeding is necessary as less than half the bleeding is related to gastric varices. SVT in acute or chronic pancreatitis results from factorial perivenous inflammation and includes intrinsic endothelial damage caused by inflammation and extrinsic damage secondary to venous pressure resulting from fibrosis, adjacent pseudocysts, or edema [3]. In any patient with signs of splenic vein thrombosis and the resulting hypersplenism, the underlying cause for hypercoagulation state should be investigated. Hypercoagulable states are inherited or acquired conditions. They are associated with a predisposition to vein thrombosis. These include a wide range of thrombolytic disorders throughout the body such as brain vein thrombosis, extremity deep venous thrombosis, arterial thrombosis (such as stroke, myocardial infarction), and intra-abdominal venous thrombosis. The most prevalent clinical feature resulting from hypercoagulable states is venous thromboembolic disease. Some other resulting disorders include myeloproliferative and hyperhomocysteinemia syndromes and antiphospholipid antibodies (APAs). As mentioned earlier our patient turned negative for MPS but in the subsequent tests blood homocysteine level was checked to be normal. APS panel was checked to show no specific underlying disorder (values are cited in Table 1) [8].

Obstruction of the splenic vein could result from enlarged retroperitoneal lymph nodes and pancreatic or perisplenic nodes [3]. These lymph nodes surround splenic veins and if they get enlarged due to inflammation or malignancy, put pressure on veins and cause obstruction and thrombosis [7]. Despite earlier reports pointing to pancreatic carcinoma as the most common cause of SVT [7, 9] most recent studies conclude that acute or chronic pancreatitis particularly in pancreatic tail is the probable cause of SVT in most patients [1, 9].

Pancreatitis is the initiating cause of thrombosis in 60% of patients; however, diagnosis of SVT in these patients does not always occur during an acute attack [3, 9]. Other causes for SVT include the following: adenopathy of metastatic carcinoma and lymphoma and iatrogenic reasons after operations such as partial gastrectomy and distal splenorenal shunt [1, 2]. Splenectomy is the treatment of choice for SVT [5]. In patients with gastric varices bleeding resulting from SVT, splenectomy must be done as an emergency because gastric varices have a relatively higher potential for massive bleeding than esophageal varices. And also no other treatment is available to put the bleeding under control [7, 10]. The role of prophylactic splenectomy in asymptomatic patients is still controversial. Previous studies showed high rates of variceal hemorrhage in patients with SVT; therefore many authors recommended splenectomy in this group of patients. But recent studies indicate that gastric varices bleeding only occurs in 40% of patients and thus prophylactic splenectomy is not recommended [7]. In this case no underlying disorders such as pancreatic associated etiologies (e.g., traumas, gastric operations, umbilical vein catheterizations, malignancies, retroperitoneal disease, splenic artery aneurysms, myeloproliferative disease, protein S deficiency, protein C deficiency, antithrombin III deficiency, and factor V Leiden mutation) were present and no evidence of antiphospholipid syndrome was seen. This convinced us that this case was interesting enough to be reported as an idiopathic isolated splenic vein thrombosis case.

## Figures and Tables

**Table 1:** Laboratory evaluation.

Beta 2 GP1 level	
IgG	4.3 AU/mL (normal: <5)
IgM	3.2 AU/mL (normal: <8)
IgA	2.4 AU/mL (normal: <8)
Anticardiolipin Ab	
IgM	9.63 MPL/mL
Lupus anticoagulant Ab	Absent
Protein C	85.6 (normal: 70%–130%)
Protein S	120.2 (normal: 77%–143%)
Antithrombin III	97% (normal: >75%)
Factor V Leiden	Absent
